# Supplementary material for: A high-resolution mRNA expression time course of embryonic development in zebrafish
Source: eLife. 2017 Nov 16;6:e30860. doi: 10.7554/eLife.30860 (PMC5690287; doi:10.7554/eLife.30860)
Supplement: Supplementary file 6. [file elife-30860-supp6.zip › biolayout-clusters-files/Cluster202-genes.html]

Cluster202


# Cluster202: Genes

| Ensembl ID | Gene Name | Chr | Start | End | Biotype |
| --- | --- | --- | --- | --- | --- |
| ENSDARG00000062190 | PDE3B | 7 | 27431120 | 27527510 | protein\_coding |
| ENSDARG00000058835 | PLEKHG3 | 20 | 3132647 | 3183121 | protein\_coding |
| ENSDARG00000076559 | RB1CC1 | 24 | 35082261 | 35117603 | protein\_coding |
| ENSDARG00000075397 | cipca | 17 | 44642482 | 44647815 | protein\_coding |
| ENSDARG00000036636 | ebpl | 1 | 45968981 | 45972765 | protein\_coding |
| ENSDARG00000059354 | glmp | 19 | 798780 | 808456 | protein\_coding |
| ENSDARG00000003087 | ubxn6 | 22 | 22235232 | 22249415 | protein\_coding |
